# Supplementary material for: The burden of malaria-attributable maternal anaemia and the impact of preventive treatment across sub-Saharan Africa
Source: Nat Health. 2026 Feb 25;1(5):497–510. doi: 10.1038/s44360-026-00068-3 (PMC13156044; doi:10.1038/s44360-026-00068-3)
Supplement: Supplementary file 1 — Supplementary Tables 1–4 and Supplementary Fig. 1. [file 44360_2026_68_MOESM1_ESM.pdf]

nature health

Supplementary information https://doi.org/10.1038/s44360-026-00068-3

The burden of malaria-attributable maternal anaemia and the impact of preventive treatment across sub-Saharan Africa

In the format provided by the authors and unedited

This Supplementary Information contains:

Supplementary Figure S1

Supplementary Table S1-4

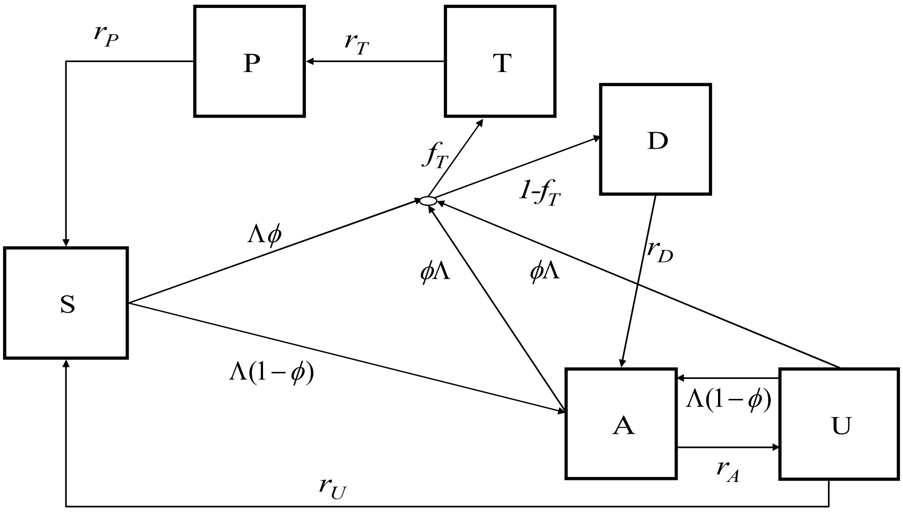

Supplementary Figure S1| Flow diagram for human stages of the general population model (taken from Griffin et al.[57].) Individuals transition between six states: susceptible (S), clinical disease untreated (D), treated disease (T), prophylaxis (P), asymptomatic infection (A), and subpatent infection (U). Susceptible individuals may acquire infection at a rate determined by the local force of infection (Λ), driven by sympatric anopheline mosquito density. Upon infection, a proportion (ϕ) develop clinical disease (D) while the remainder $(1-\phi)$ experience asymptomatic infection (A). Clinical cases may be treated (probability f_T) and move to the treated state (T), or remain untreated and transition to asymptomatic infection. Treated individuals pass into a post-treatment prophylaxis state (P), after which they return to susceptibility. Asymptomatic infections may progress to subpatent infection (U), or revert to clinical disease upon reinfection. Subpatent infections may either clear naturally and return to susceptibility or revert to asymptomatic infection following reinfection. All infected states are subject to clearance at rates (r_D, r_T, r_A, r_U, r_P) specific to each pathway. The probability a new infection is symptomatic (ϕ) and the clearance rate of asymptomatic infection (r_A) are immunity-dependent, with immunity increasing with age and cumulative prior malaria exposure; consequently, ϕ decreases and r_A increases with higher immunity.

Supplementary Table S1: Study Characteristics for Hemoglobin–Malaria Association Model

Trial name and country	Malawi IST	Kenya IST	West Africa IST: Burkina Faso, Mali, Gambia, and Ghana	IMPROVE-1: Kenya, Malawi, and Tanzania
Trial arms	ISTp-DP v IPTp-SP	ISTp-DP v. IPTp-DP v IPTp-SP	ISTp-AL v IPTp-SP	IPTp-DP v IPTp-DP+AZ v IPTp-SP
Reference	Madanitsa et al. 2016[24]	Desai et al, 2015[25]	Tagbor et al, 2015[27]	Madanitsa et al, 2023[26]
Study identifier	ISRCTN: 69800930	Clinicaltrials.gov registration number NCT01669941	Clinicaltrials.gov registration number: NCT01084213	Clinicaltrials.gov registration number: NCT03208179
Timing of trial	July 21, 2011 – March 18, 2013	Aug 21, 2012 – June 19, 2014	May 31, 2010 – Oct 31, 2011	March 29, 2018 – July 5, 2019
Study locations	Southern Region, Malawi	Siaya county, Western Kenya	Plateau-Central, Burkina Faso, Southern Mali (Kita, Yirimadjom and San), Basse, The Gambia and Navrongo, Ghana	Western Kenya: (Kisumu, Siaya and Homa Bay) Malawi: Southern Region, Tanzania: Tanga region
Malaria transmission	Moderate to intense year-round malaria transmission	Intense year-round malaria transmission	Moderately high or high and seasonal	Perennial malaria transmission
Number of participants (included in model fitting*)	1873 (1803)	1546 (1396)	5354 (5208, Burkina Faso: 1413, Gambia: 1193, Ghana: 1298, Mali: 1304)	4680 (4201, Kenya: 1297, Malawi: 1283, Tanzania: 1621)
Gestational age at enrollment (per protocol)	16-28 weeks	16-32 weeks	16-30 weeks	16-28 weeks
Gravidity recruited	All	All	G1 and G2	All
Gestational age measurement	Ultrasound	LMP and physical examination	Symphysis-fundal height	Ultrasound
Anemia at enrollment exclusion criteria	<7 g/dL	<7 g/dL	Not excluded	Not excluded
PCR prevalence at enrollment in primigravidae	58.5%	40.3%	Burkina Faso: 65.1%<br>The Gambia: 13.9%<br>Ghana: 63.6%<br>Mali: 37.1%	Kenya: 23.4%<br>Malawi: 27.9%<br>Tanzania:12.9%

**Excluded women for missing information on gestational age, PCR malaria infection, gravidity, or hemoglobin concentration at enrollment. Abbreviations: Azithromycin (AZ).*

Supplementary Table S2: Model comparison based on Deviance Information Criterion (DIC)

Trajectory with gestational age in absence of malaria	Malaria impact (with gestational age)	Gravidity-dependent in absence of malaria	Pregnancy-specific immunity	DIC
Constant	None	No	No	62620.8
Constant	Constant	No	No	61984.4
Constant	Cubic spline	No	No	61940.8
Cubic spline	Constant	No	No	61790.4
Cubic spline	Cubic spline	No	No	61794.0
Cubic spline	Cubic spline	Yes	No	61568.5
Cubic spline	Cubic spline	No	Yes	61539.1
Cubic spline	Constant	Yes	Yes	61510.3
Cubic spline	Cubic spline	Yes	Yes	61497.5

DIC values for Hb trajectory models differ by specification of gestational age effects, malaria-associated reductions, and inclusion of gravidity- and immunity-dependent modifiers. Lower DIC indicates better model fit.

Supplementary Table S3: Parameters of the final fitted model with associated prior and posterior distributions

Parameter	Symbol	Prior	Posterior Median (95% CrI)
**Core anemia model parameters**			
Cubic spline of impact of gestational age upon Hb (first knot)	α_1	$U(-15,15)$	11.73 (11.49, 11.98)
Cubic spline of impact of gestational age upon Hb g/dL (second knot)	α_2		10.98 (10.89, 11.07)
Cubic spline of impact of gestational age upon Hb g/dL (third knot)	α_3		10.55 (10.39, 10.71)
Cubic spline of malaria impact on Hb g/dL with gestational age (first knot)	β_1	$U(-15,15)$	-1.31 (-1.86, -0.75)
Cubic spline of malaria impact on Hb g/dL with gestational age (second knot)	β_2		-1.38 (-1.51, -1.25)
Cubic spline of malaria impact on Hb g/dL with gestational age (third knot)	β_3		-1.82 (-2.22, -1.41)
Standard Deviation of Hb g/dL	σ	$\Gamma(0.01,0.01)$	1.45 (1.42, 1.47)
Non-malaria associated gravidity impact (G2 vs G1)	γ_2	$U(-5,5)$	0.21 (0.13, 0.29)

Non-malaria associated gravidity impact (G3 vs G1)	γ_3		0.36 (0.25, 0.48)
Non-malaria associated gravidity impact (G4 vs G1)	γ_4		0.30 (0.15, 0.45)
Non-malaria associated gravidity impact (G5 vs G1)	γ_5		0.05 (-0.12, 0.21)
Non-malaria associated gravidity impact (G6+ vs G1)	γ_6		0.24 (0.13, 0.35)
Impact of pregnancy-specific immunity (shape parameter)	κ	$\Gamma(2,1)$	5.77 (1.54, 9.92)
Impact of pregnancy-specific immunity (scale parameter)	ν	$\Gamma(2,1)$	0.83 (0.42, 0.93)
**Fixed effects: difference in mean Hb relative to equivalent individual in Malawi IST trial**			
Burkina Faso (West Africa IST trial)	δ_s	$U(-15,15)$	-0.19 (-0.32, -0.06)
The Gambia (West Africa IST trial)			-0.49 (-0.63, -0.35)
Ghana (West Africa IST trial)			-0.49 (-0.63, -0.35)
Mali (West Africa IST trial)			-0.02 (-0.16, 0.11)
Kenya (IMPROVE-1 trial)			-0.10 (-0.21, 0.01)
Malawi (IMPROVE-1 trial)			0.28 (0.18, 0.39)
Tanzania (IMPROVE-1 trial)			-0.03 (-0.13, 0.07)
Kenya (IST trial)			-0.37 (-0.49, -0.26)
**Parameters determining PCR prevalence (log odds of infection) in primigravidae by site**			
Burkina Faso (West Africa IST trial)	o_{s1}	$U(-4.6,1.736)$	0.59 (0.36, 0.81)
The Gambia (West Africa IST trial)			-1.82 (-2.14, -1.52)
Ghana (West Africa IST trial)			0.54 (0.32, 0.77)
Mali (West Africa IST trial)			-0.56 (-0.78, -0.36)
Kenya (IMPROVE-1 trial)			-1.20 (-1.42, -0.99)
Malawi (IMPROVE-1 trial)			-0.94 (-1.17, -0.73)
Tanzania (IMPROVE-1 trial)			-1.89 (-2.17, -1.62)
Kenya (IST trial)			-0.15 (-0.35, 0.04)
Malawi (IST trial)			0.51 (0.35, 0.67)
**Additional Parameters to allow for adjustment according to censoring (Kenya IST trial)**			

Gravidity pattern relative risk (G2 vs G1)	RR_{s2}	$U(-10,10)$	-0.61 (-0.77, -0.46)
Gravidity pattern relative risk (G3 vs G1)	RR_{s3}		-0.75 (-0.92, -0.59)
Gravidity pattern relative risk (G4 vs G1)	RR_{s4}		-0.98 (-1.16, -0.80)
Gravidity pattern relative risk (G5 vs G1)	RR_{s5}		-1.66 (-1.89, -1.43)
Gravidity pattern relative risk (G6+ vs G1)	RR_{s6}		-1.44 (-1.66, -1.24)
Log odds of infection (G2)	o_{s2}	$U(-4.6,1.736)$	-0.67 (-0.95, -0.41)
Log odds of infection (G3)	o_{s3}		-0.94 (-1.26, -0.64)
Log odds of infection (G4)	o_{s4}		-1.14 (-1.51, -0.81)
Log odds of infection (G5)	o_{s5}		-0.92 (-1.40, -0.47)
Log odds of infection (G6+)	o_{s6}		-1.14 (-1.61, -0.71)
Distribution of gestational age at enrollment (first shape parameter)	λ_{s1}	$\Gamma(0.01,0.01)$	1.95 (1.80, 2.10)
Distribution of gestational age at enrollment (second shape parameter)	λ_{s2}	$\Gamma(0.01,0.01)$	1.30 (1.20, 1.40)
**Additional Parameters to allow for adjustment according to censoring (Malawi IST trial)**			
Gravidity pattern relative risk (G2 vs G1)	RR_{s2}	$U(-10,10)$	-0.28 (-0.46, -0.10)
Gravidity pattern relative risk (G3 vs G1)	RR_{s3}		-0.85 (-1.10, -0.60)
Gravidity pattern relative risk (G4 vs G1)	RR_{s4}		-0.71 (-0.97, -0.46)
Gravidity pattern relative risk (G5 vs G1)	RR_{s5}		-0.92 (-1.32, -0.54)
Log odds of infection (G2)	o_{s2}	$U(-4.6,1.736)$	-0.31 (-0.43, -0.19)
Log odds of infection (G3)	o_{s3}		-0.84 (-0.98, -0.71)
Log odds of infection (G4)	o_{s4}		-0.98 (-1.13, -0.84)
Log odds of infection (G5)	o_{s5}		-1.70 (-1.89, -1.52)
Distribution of gestational age at enrollment (first shape parameter)	λ_{s1}	$\Gamma(0.01,0.01)$	3.58 (3.36, 3.82)
Distribution of gestational age at enrollment (second shape parameter)	λ_{s2}	$\Gamma(0.01,0.01)$	2.88 (2.70, 3.07)

Table S4. Summary of IPTp-SP Trials Assessing Impact on Hemoglobin Concentration

Study	Location and Date	IPTp Regimen	Gravidity Category	Result: Mean Hb Difference (SP – Placebo)	Prevalence in Placebo Arm	Included in Main Analysis?
**Mbaye et al, 2006**[35]	Farafenni Gambia, 2002–2004	Monthly SP (up to 4 doses)	G2+	-0.10 (-0.45–0.25)	15.0% SM at enrollment	No – excluded severely anemic (Hb <7 g/dL)
**Ndyomugyenyi et al, 2011** [37]	Kabale, Uganda, 2004–2007	Two-dose SP	All women	0.06 (-0.08–0.20)	10.3% SM (third trimester, LLIN only arm)	No – excluded severely anemic (Hb <7 g/dL)
**Njagi et al, 2003**[36]	Western Kenya, 1997–1998	Two-dose SP	G1	0.68 (0.10–1.26)	47.8% SM in G1 at enrollment	Yes
	Western Kenya, 1997–1998	Two-dose SP + LLIN vs LLIN	G1	0.26 (-0.24–0.76)	47.8% SM in G1 at enrollment	No – different intervention
	Western Kenya, 1997–1998	Two-dose SP	G2	0.69 (0.07–1.31)	47.8% SM in G1 at enrollment	Yes
	Western Kenya, 1997–1998	Two-dose SP + LLIN vs LLIN	G2	0.12 (-0.53–0.77)	47.8% SM in G1 at enrollment	No – different intervention
**Parise et al, 1998** [33]	Western Kenya, 1994–1996	Two-dose SP	G1–2	0.30 (0.01–0.59)	44.9% SM (G1 and G2) at enrollment	Yes
	Western Kenya, 1994–1996	Monthly SP	G1–2	0.50 (0.20–0.80)	44.9% SM (G1 and G2) at enrollment	No – different intervention
**Shulman et al (1999)**[34]	Kilifi, Kenya, 1996–1997	Three-dose SP	G1	0.40 (0.19–0.61)	35.3% SM in T3 (placebo arm)	Yes

Abbreviations: SM (Slide microscopy), LLIN (long lasting insecticidal net), T3 – third trimester
