# Supplementary material for: The burden of malaria-attributable maternal anaemia and the impact of preventive treatment across sub-Saharan Africa
Source: Nat Health. 2026 Feb 25;1(5):497–510. doi: 10.1038/s44360-026-00068-3 (PMC13156044; doi:10.1038/s44360-026-00068-3)
Supplement: Supplementary file 2 — Reporting Summary [file 44360_2026_68_MOESM2_ESM.pdf]

nature portfolio

Corresponding author(s): Patrick Walker

Last updated by author(s): Aug 5, 2025

Reporting Summary

Nature Portfolio wishes to improve the reproducibility of the work that we publish. This form provides structure for consistency and transparency in reporting. For further information on Nature Portfolio policies, see our Editorial Policies and the Editorial Policy Checklist.

Statistics

For all statistical analyses, confirm that the following items are present in the figure legend, table legend, main text, or Methods section.

n/a	Confirmed	
☐	☒	The exact sample size (n) for each experimental group/condition, given as a discrete number and unit of measurement
☐	☒	A statement on whether measurements were taken from distinct samples or whether the same sample was measured repeatedly
☐	☒	The statistical test(s) used AND whether they are one- or two-sided<br>*Only common tests should be described solely by name; describe more complex techniques in the Methods section.*
☐	☒	A description of all covariates tested
☐	☒	A description of any assumptions or corrections, such as tests of normality and adjustment for multiple comparisons
☐	☒	A full description of the statistical parameters including central tendency (e.g. means) or other basic estimates (e.g. regression coefficient) AND variation (e.g. standard deviation) or associated estimates of uncertainty (e.g. confidence intervals)
☒	☐	For null hypothesis testing, the test statistic (e.g. F, t, r) with confidence intervals, effect sizes, degrees of freedom and P value noted<br>*Give P values as exact values whenever suitable.*
☐	☒	For Bayesian analysis, information on the choice of priors and Markov chain Monte Carlo settings
☐	☒	For hierarchical and complex designs, identification of the appropriate level for tests and full reporting of outcomes
☒	☐	Estimates of effect sizes (e.g. Cohen's d, Pearson's r), indicating how they were calculated

Our web collection on statistics for biologists contains articles on many of the points above.

Software and code

Policy information about availability of computer code

Data collection	Our analysis involved no primary data collection.
Data analysis	All code used for this analysis is open access and available via the following repositories:<br><br>Hemoglobin dynamics and anemia risk model: https://github.com/patrickgtwalker/Anaemia_malaria_fitting_open<br><br>Integrated malaria in pregnancy transmission model: https://github.com/patrickgtwalker/malaria_in_pregnancy_istp_model_open<br><br>Both repositories include full documentation and implementation guidance. These resources provide all code necessary to reproduce the main analyses and figures. Feedback and contributions are welcome.

For manuscripts utilizing custom algorithms or software that are central to the research but not yet described in published literature, software must be made available to editors and reviewers. We strongly encourage code deposition in a community repository (e.g. GitHub). See the Nature Portfolio guidelines for submitting code & software for further information.

Data

Policy information about availability of data

All manuscripts must include a data availability statement. This statement should provide the following information, where applicable:

- Accession codes, unique identifiers, or web links for publicly available datasets
- A description of any restrictions on data availability
- For clinical datasets or third party data, please ensure that the statement adheres to our policy

The dataset from Tagbor et al. (2015) is archived at the LSHTM Data Compass repository (DOI: 10.17037/DATA.2) and is available under controlled access upon request.

The dataset from Madanitsa et al. (2016) is available via the WorldWide Antimalarial Resistance Network (WWARN), with access subject to review by the WWARN Data Access Committee.

Individual participant-level data from Desai et al. (2015) and Madanitsa et al. (2023) are available from the study investigators upon reasonable request and subject to appropriate data use agreements and ethical approvals.

All processed data outputs used to generate model results (e.g., hemoglobin trajectories, anemia risk estimates, IPTp impact) are included in the Supplementary Information. All secondary data sources—including malaria prevalence surfaces (Malaria Atlas Project), demographic and fertility data (DHS/MIS), and population estimates (WorldPop)—are publicly available and cited in the manuscript.

Research involving human participants, their data, or biological material

Policy information about studies with human participants or human data. See also policy information about sex, gender (identity/presentation), and sexual orientation and race, ethnicity and racism.

Reporting on sex and gender	All trials enrolled pregnant individuals and included data on gravidity (parity), but not on gender identity or presentation. All participants were biologically female by study design.
Reporting on race, ethnicity, or other socially relevant groupings	Race and ethnicity were not recorded in any of the trials and were not used in the design, analysis, or interpretation of the study. Country of residence and site-specific malaria risk were used to capture geographic and epidemiologic heterogeneity.
Population characteristics	The study population comprises pregnant women attending antenatal care across sites in Burkina Faso, Ghana, The Gambia, Mali, Kenya, Malawi, and Tanzania between 2010 and 2019. Participants were enrolled during the second trimester (14–27 weeks' gestation) as part of four randomized controlled trials of malaria prevention in pregnancy and followed through delivery.
Recruitment	Participants were recruited through antenatal clinics in the context of ethically approved clinical trials. All participants provided written informed consent prior to enrollment. Trial-specific eligibility criteria, randomization procedures, and clinical endpoints are detailed in the original publications.
Ethics oversight	This study is a secondary analysis of de-identified individual-level data from four previously published randomized trials. Each trial was approved by the relevant national research ethics committees and institutional review boards in participating countries. Written informed consent was obtained from all participants at the time of enrollment. This secondary analysis was conducted with the permission of the original trial investigators and adheres to all applicable ethical and data protection standards. No new participants were enrolled, and no additional data collection was undertaken.

Note that full information on the approval of the study protocol must also be provided in the manuscript.

Field-specific reporting

Please select the one below that is the best fit for your research. If you are not sure, read the appropriate sections before making your selection.

☒ Life sciences ☐ Behavioural & social sciences ☐ Ecological, evolutionary & environmental sciences

For a reference copy of the document with all sections, see nature.com/documents/nr-reporting-summary-flat.pdf

Life sciences study design

All studies must disclose on these points even when the disclosure is negative.

Sample size	No new participants were enrolled for this secondary analysis. The sample size (n = 12,608) reflects the total number of pregnant women enrolled in four previously conducted randomized controlled trials in sub-Saharan Africa. Sample size in each trial was predetermined based on the original clinical objectives and statistical power calculations as described in the trial protocols and primary publications. All available participant data from these trials were included in the analysis.
Data exclusions	No additional exclusion criteria were applied beyond those specified in the original trial protocols. In some trials, exclusions were applied for biological or protocol-driven reasons—such as restriction to specific gravidity groups or exclusion of women with hemoglobin <7 g/dL at

enrollment due to concerns about trial safety. Our analytic approach explicitly adjusted for these exclusions to ensure that the resulting estimates are representative of the broader population of antenatal care attendees. Full details of the trial-specific exclusion criteria and how they were accounted for in the analysis are provided in the Methods and Supplementary Information.

Replication

This study used previously published datasets and did not include experimental replication. Reproducibility of the findings was evaluated by testing model fit across multiple trials and sites, as well as by reproducing observed hemoglobin trajectories and anemia prevalence patterns. All code used for model fitting and replication is available in public GitHub repositories.

Randomization

All participants included in the analysis were originally enrolled in randomized controlled trials. Randomization procedures—including group allocation methods and control of potential confounders—were determined by each trial's design and are detailed in their original publications.

Blinding

Blinding of participants and study staff was implemented in the original trials according to their respective protocols (e.g., double-blind, placebo-controlled designs).

Reporting for specific materials, systems and methods

We require information from authors about some types of materials, experimental systems and methods used in many studies. Here, indicate whether each material, system or method listed is relevant to your study. If you are not sure if a list item applies to your research, read the appropriate section before selecting a response.

Materials & experimental systems

n/a	Involved in the study
☒	☐ Antibodies
☒	☐ Eukaryotic cell lines
☒	☐ Palaeontology and archaeology
☒	☐ Animals and other organisms
☐	☒ Clinical data
☒	☐ Dual use research of concern
☒	☐ Plants

Methods

n/a	Involved in the study
☒	☐ ChIP-seq
☒	☐ Flow cytometry
☒	☐ MRI-based neuroimaging

Clinical data

Policy information about clinical studies

All manuscripts should comply with the ICMJE guidelines for publication of clinical research and a completed CONSORT checklist must be included with all submissions.

Clinical trial registration

This study is a secondary analysis of de-identified individual-level data from four randomized controlled trials of malaria in pregnancy, all prospectively registered:
Tagbor et al. (2015) — NCT01084213
Desai et al. (2015) — NCT01669941
Madanitsa et al. (2016) — Pan AfricanClinical Trials Registry PACTR201103000280319;ISRCTNRegistry ISRCTN69800930
Madanitsa et al., (2023) — NCT03208179
Each trial was conducted under ethical oversight by recognized national and institutional review boards.

Study protocol

This study involved secondary analysis of anonymized individual participant data from four randomized controlled trials of malaria prevention in pregnancy. All trials enrolled pregnant women through antenatal care (ANC) clinics in sub-Saharan Africa and recorded hemoglobin concentration, malaria status, gestational age, and gravidity at enrollment. No new data collection or participant enrolment was conducted for this analysis. Trial-specific inclusion and exclusion criteria were accounted for to ensure population representativeness.

Madanitsa et al. (2023): A double-blind, randomized, partly placebo-controlled trial conducted in Kenya, Malawi, Tanzania, and Burkina Faso. Women aged ≥15 years at 16–28 weeks' gestation were randomized to monthly sulfadoxine–pyrimethamine (SP), dihydroartemisinin–piperaquine (DP), DP with azithromycin (AZ), or placebo (in selected sites). Key exclusion criteria included multiple pregnancy, chronic illness, and contraindications to study drugs. Women were not excluded based on hemoglobin concentration.

Madanitsa et al. (2016): A randomized trial in southern Malawi comparing monthly IPTp with SP versus DP. Participants were enrolled via ANC and followed through delivery. Women with Hb <7 g/dL were excluded at baseline.

Desai et al. (2015): An open-label, three-arm randomized superiority trial conducted in western Kenya. Women at 16–32 weeks' gestation were randomized to IPTp-SP, ISTp-DP, or IPTp-DP. Primary outcomes included maternal malaria and anemia. Women with Hb <7 g/dL were excluded.

Tagbor et al. (2015): A three-arm randomized controlled non-inferiority trial conducted in Burkina Faso, Mali, The Gambia, and Ghana. Women were randomized to IPTp-SP, ISTp-SP, or ISTp-AS–AQ and followed monthly through delivery. The trial enrolled only primigravidae and secundigravidae. No exclusion based on hemoglobin concentration was applied.

All original trials received ethics approval from local and collaborating institutional review boards, and written informed consent was obtained from all participants at the time of enrolment.

Data collection

Individual-level data were obtained from four randomized controlled trials conducted between 2010 and 2019 in seven sub-Saharan African countries. All data used in this analysis were collected at the point of enrolment during antenatal care (ANC) visits, prior to administration of intermittent preventive treatment. Key variables included hemoglobin concentration, malaria infection status (by PCR or microscopy), gestational age, and gravidity.
Madanitsa et al., 2016 (Malawi IST): Enrolment took place between 2011 and 2013 in southern Malawi. Women were enrolled at 14–26 weeks' gestation.
Desai et al., 2015 (Kenya IST): Conducted in western Kenya from 2012 to 2014. Women were enrolled at 16–32 weeks' gestation.
Tagbor et al., 20105(West Africa trial): Participants were enrolled between 2010 and 2011 in Ghana, Burkina Faso, Mali, and The Gambia. Only primigravidae and secundigravidae were eligible.No gestational limts on enrollment were applied.
Madanitsa et al., 2023 (IMPROVE-1): Enrolment occurred between 2018 and 2019 in Kenya, Malawi, and Tanzania. Women were enrolled at 16–28 weeks' gestation.

Outcomes

The primary outcome for our secondary analysis was the prevalence of malaria-attributable maternal anemia, stratified by severity (moderate: Hb <9 g/dL; severe: Hb <7 g/dL) and gestational age. These thresholds were pre-specified based on established clinical risk levels for postpartum hemorrhage and maternal mortality. Hemoglobin concentration at enrolment was recorded using standard clinical protocols in all four source trials. Malaria status was determined via PCR (or microscopy where PCR was unavailable), and gestational age was based on last menstrual period or fundal height as recorded at enrolment.

Plants

Seed stocks

Report on the source of all seed stocks or other plant material used. If applicable, state the seed stock centre and catalogue number. If plant specimens were collected from the field, describe the collection location, date and sampling procedures.

Novel plant genotypes

Describe the methods by which all novel plant genotypes were produced. This includes those generated by transgenic approaches, gene editing, chemical/radiation-based mutagenesis and hybridization. For transgenic lines, describe the transformation method, the number of independent lines analyzed and the generation upon which experiments were performed. For gene-edited lines, describe the editor used, the endogenous sequence targeted for editing, the targeting guide RNA sequence (if applicable) and how the editor was applied.

Authentication

Describe any authentication procedures for each seed stock used or novel genotype generated. Describe any experiments used to assess the effect of a mutation and, where applicable, how potential secondary effects (e.g. second site T-DNA insertions, mosiacism, off-target gene editing) were examined.
